# Antimicrobial Photodynamic Therapy Involving a Novel Photosensitizer Combined With an Antibiotic in the Treatment of Rabbit Tibial Osteomyelitis Caused by Drug-Resistant Bacteria

**DOI:** 10.3389/fmicb.2022.876166

**Published:** 2022-04-22

**Authors:** Xiujuan Yin, Ziyuan Fang, Yan Fang, Lin Zhu, Jinwen Pang, Tianjun Liu, Zhanjuan Zhao, Jianxi Zhao

**Affiliations:** ^1^School of Clinical Medicine, Hebei University, Baoding, China; ^2^Tianjin Key Laboratory of Biomedical Material, Institute of Biomedical Engineering, Peking Union Medical College, Chinese Academy of Medical Sciences, Tianjin, China; ^3^School of Basic Medicine, Hebei University, Baoding, China; ^4^Department of Radiology, Affiliated Hospital of Hebei University, Baoding, China

**Keywords:** antibiotic resistance, photodynamic antibacterial chemotherapy, photosensitizer, osteomyelitis, antimicrobial effect

## Abstract

Osteomyelitis is deep tissue inflammation caused by bacterial infection. If such an infection persists, it can lead to dissolution and necrosis of the bone tissue. As a result of the extensive use of antibiotics, drug-resistant bacteria are an increasingly common cause of osteomyelitis, limiting the treatment options available to surgeons. Photodynamic antibacterial chemotherapy has attracted increasing attention as a potential alternative treatment. Its advantages are a broad antibacterial spectrum, lack of drug resistance, and lack of toxic side effects. In this study, we explored the impact of the new photosensitizer LD4 in photodynamic antimicrobial chemotherapy (PACT), both alone and in combination with an antibiotic, on osteomyelitis. A rabbit tibial osteomyelitis model was employed and microbiological, histological, and radiological studies were performed. New Zealand white rabbits (*n* = 36) were randomly divided into a control group, antibiotic group, PACT group and PACT + antibiotic group for treatment. In microbiological analysis, a reduction in bacterial numbers of more than 99.9% was recorded in the PACT group and the PACT + antibiotic group 5 weeks after treatment (*p* < 0.01). In histological analysis, repair of the damaged bone tissue was observed in the PACT group, and bone repair in the PACT + antibiotic group was even more significant. In radiological analysis, the X-ray Norden score showed that the severity of bone tissue defects or destruction followed the pattern: PACT + antibiotic group < PACT group < antibiotic group < control group.

## Introduction

Infecting microorganisms can lead to the progressive destruction and necrosis of bone tissue, known as osteomyelitis (Lew and Waldvogel, 2004). Osteomyelitis can be divided into three subtypes: acute, subacute, and chronic. Acute and subacute osteomyelitis mainly occur in children and older people (Kavanagh et al., 2018). Chronic osteomyelitis is common in open fractures, diabetes, and bone infections caused by drug-resistant bacteria (Dudareva et al., 2019), and about 20% of diabetic foot patients experience osteomyelitis (Geraghty and Laporta, 2019). Osteomyelitis can lead to bone defects and osteonecrosis, with a high recurrence rate. Pathogenic bacteria that are known causative agents of osteomyelitis include *Staphylococcus aureus*, *Pseudomonas aeruginosa*, and *Escherichia coli* (Yang et al., 2020). *Staphylococcus aureus* accounts for about 75% of the pathogenic bacteria responsible for osteomyelitis (Thorpe et al., 2018). This microorganism is one of the most drug-resistant pathogenic bacteria, and a common drug-resistant strain is methicillin-resistant *S. aureus* (MRSA). *Staphylococcus aureus* can effectively protect itself from the external environment by altering peptidoglycan synthesis and the composition of its cell wall, and by biofilm formation (Archer et al., 2011). The increase in prevalence of drug-resistant strains, particularly the increase in drug-resistant *S. aureus*, makes treating osteomyelitis or bone infection ever more challenging (Johnson et al., 2018). It is estimated that by 2050, nearly 10 million people may die from drug-resistant strains every year (Sutradhar et al., 2021).

At present, the treatment methods available for osteomyelitis mainly include surgical debridement and antibiotic treatment (Niikura et al., 2016). Surgical debridement to remove residual pathogenic bacteria in the medullary cavity is technically challenging (Surdu-Bob et al., 2016). Alternatively, the extensive and large-dose application of antibiotics may lead to the emergence of multidrug-resistant strains, further exacerbating the complexity of treating osteomyelitis caused by drug-resistant strains (Sheehy et al., 2010). At present, osteomyelitis as associated with a slow treatment process and a low cure rate. Patients with severe illness also face the risk of amputation (Tschudin-Sutter et al., 2016). Therefore, new treatments with sound curative effects, few side effects, and reduced recurrence rates are urgently needed. Photodynamic antimicrobial chemotherapy (PACT) has gradually attracted increasing attention because of its minor toxicity and side effects, lack of drug resistance, and wide antibacterial spectrum (Tavares et al., 2010).

The principle of PACT is that photosensitizers with no toxicity or side effects are first specifically aggregated in bacteria. Then, the bacteria are irradiated with visible light at an appropriate wavelength to activate the photosensitizers in bacteria to produce reactive oxygen species (ROS), which are cytotoxic and inactivate the bacteria (Wainwright, 1998). In recent years, its clinical applications have expanded from malignant tumors to more benign diseases. PACT is considered an effective method to inactivate various microorganisms, and it exerts a sterilization effect on drug-resistant bacteria and bacterial biofilm. Because of its robust targeting, low toxicity and side effects, broad antibacterial spectrum, quick response, repeatable treatment, and lack of drug resistance, it has been widely used to treat superficial localized infections (Calabro et al., 2013; Mahmoudi et al., 2018; Huang et al., 2020). *In vitro* (Elzorkany et al., 2019; Choi et al., 2020) and *in vivo* (Mai et al., 2017; Lu et al., 2019) studies show that PACT has anti-infection characteristics and can promote wound healing. Meta-analysis of PACT shows that it is effective in promoting tissue repair, increasing the deposition of collagen in wounds, and accelerating wound healing (Garcia et al., 2010). There are many reports on the treatment of superficial infections by PACT, but few on its application in the treatment of osteomyelitis, and only one study on the combination of PACT and antibiotics in the treatment of osteomyelitis (Lu et al., 2019). To date, the treatment of osteomyelitis by PACT has not been fully applied in clinic.

Photosensitive compounds play an essential role as energy carriers and also function to bridge reactions in the treatment of osteomyelitis. Ideal photosensitive drugs should have the characteristics of high efficacy, low toxicity, good water solubility, and robust targeting. At present, photosensitizers used in photodynamic therapy of osteomyelitis mainly include 5-aminolevulinic acid (5-ALA; Bisland et al., 2006) and phenothiazines (Tardivo and Baptista, 2009; Dos Reis et al., 2015). Traditional phenothiazine dyes, such as methylene blue, toluidine blue, and other cationic photosensitizers, showed high antibacterial activity in trauma and oral infections (Ishiwata et al., 2021; Wiench et al., 2021). However, because of the unnatural origin of such photosensitizers, their biological metabolic pathway is not clear and toxicity is high, which restricts their use. 5-ALA is widely used in the clinic, but when activated by ALA dehydratase and converted into protoporphyrin ix, its concentration is relatively low, its distribution is uneven, and its photodynamic reaction efficiency is relatively low (Maisch et al., 2011).

We designed and synthesized a series of amino tetraphenylporphyrin compounds modified by basic amino acids, all of which showed good physical and chemical properties (Meng et al., 2015). Among them, compound LD4 showed high water solubility, low toxicity, and effective targeting, and thereby demonstrated potential for the specific inhibition of microbial pathogens in treating infectious diseases (Xu et al., 2016; Zhao et al., 2021). Compared with other photosensitizers, LD4 has a wider photo-inactivation range (Tardivo and Baptista, 2009; Mbakidi et al., 2013; Dos Reis et al., 2015), and higher biocompatibility and stability (Mbakidi et al., 2013; Prasanth et al., 2014). It can effectively kill gram-positive and gram-negative bacteria (Meng et al., 2015), inhibit the secretion of inflammatory factors, and promote wound healing after infection (Zhao et al., 2021). Previous experiments showed that LD4 exerted good sterilization effects on MRSA, *E. coli* and *P. aeruginosa in vitro* (Meng et al., 2015). *In vivo* experiments showed that LD4-mediated PACT had a good anti-infection effect against a mixed wound infection in rats caused by three bacteria (Xu et al., 2016).

In treating osteomyelitis, systemic use of antibiotics leads to a low concentration of antibiotics in the infected areas, which hinders the complete inactivation of pathogenic bacteria (Rao et al., 2011). Therefore, an additional benefit of PACT is that it is targeted directly to the infected area and therefore more likely to achieve bacterial eradication. In this study, PACT combined with an antibiotic was used to treat osteomyelitis by targeting infection both locally (inside the medullary cavity) and systemically (outside of the medullary cavity). PACT directly acted on the infected area, inactivated the pathogenic bacteria in the infected focus, and injected a small amount of antibiotic systemically to inhibit bacteria throughout the body. The principal aim of this study was to evaluate the effect of LD4-PACT combined with an antibiotic on rabbit tibial osteomyelitis to provide new insight into this potential clinical treatment for osteomyelitis.

## Materials and Methods

### Chemicals and Drugs

**Figure 1:**
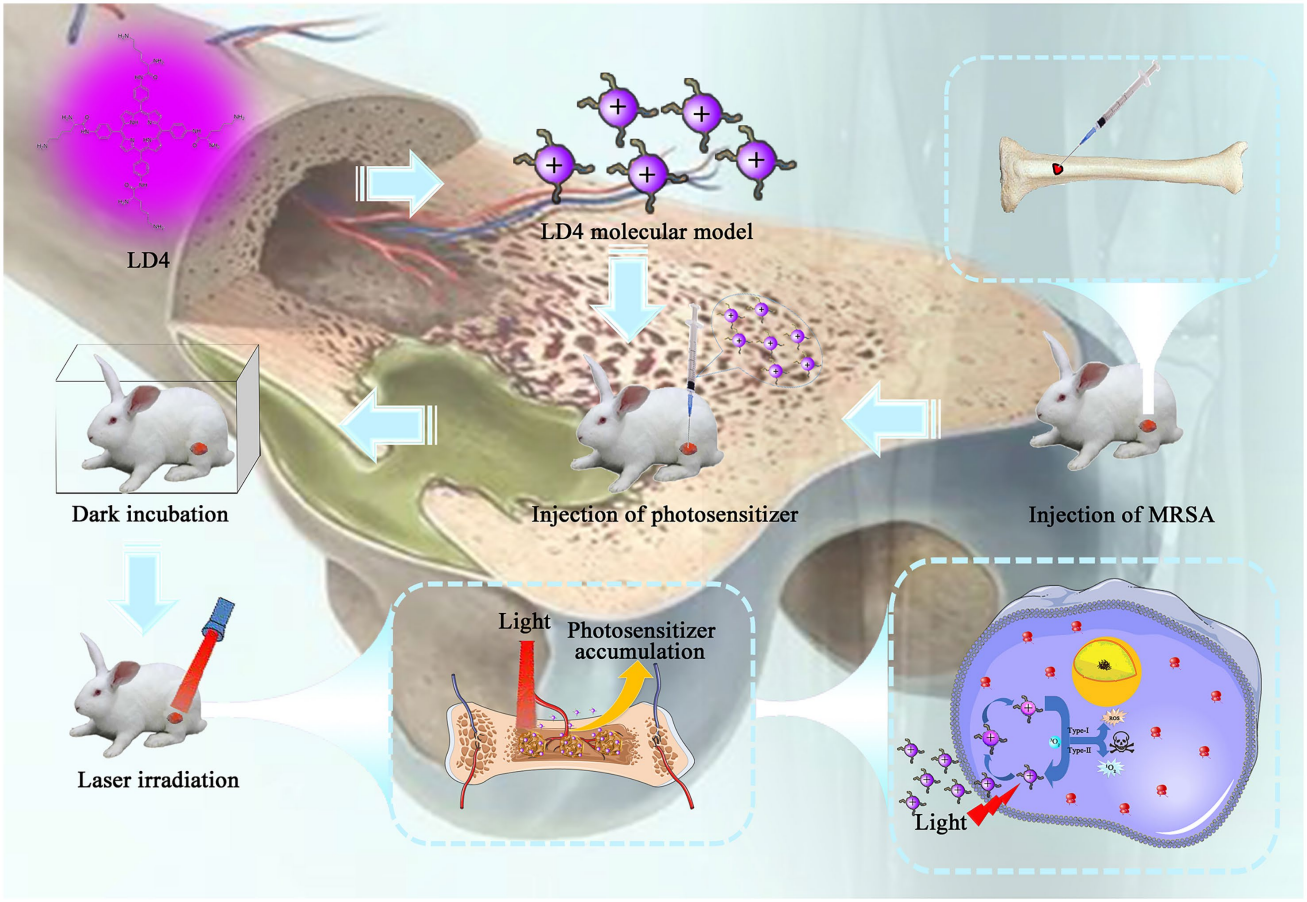
The process and mechanism of LD4-PACT treating rabbit tibial osteomyelitis.

Synthesis and characterization of 4i was previously reported by the Key Laboratory of Biomedical Material, Institute of Biomedical Engineering, Peking Union Medical College, and the Chinese Academy of Medical Sciences (Meng et al., 2015). The chemical structure of 4i is shown in Figure 1. A stock solution (500 μM) was prepared by dissolution in dimethylsulfoxide (DMSO) and was stored at −20°C in the dark before use. Gentamicin (GM) is a broad-spectrum antibiotic, which was provided by Shanghai Pharmaceutical Group (Shanghai, China).

### Light Source

A semiconductor 650 nm laser (WSLS-650-500m-200 M-H4; Wave Spectrum Laser; China) was selected for this study, and the light spot energy density was determined with an optical power meter (LM1, Carl Zeiss, Germany). The energy density of the spot was 158 mW/cm^2^, and the total energy was 95 J/cm^2^ after laser irradiation for 10 min.

### Bacterial Strain

The methicillin-resistant *S. aureus* (MRSA) strain was provided by the Laboratory Department of Affiliated Hospital of Hebei University, and was insensitive to penicillin antibiotics and sensitive to gentamicin. The antimicrobial spectrum of drug-resistant *S. aureus* is shown in Supplementary Table S1.

For culturing, a single colony of *S. aureus* was used to inoculate 5 ml of LB medium, which was then incubated at 37°C with shaking at 200 rpm, overnight. Next, 0.5 ml of the *S. aureus* bacterial culture was added to 9.5 ml of fresh culture medium, and the OD value was measured hourly with a microplate reader. Colony counts of viable bacteria were determined using the dilution plating method. The complementary linear relationship between the colony concentration and the OD value was calculated, and it was found that when OD_600_ nm = 0.5, the corresponding bacterial solution concentration was 1.5 × 10^8^ CFU/ml. This concentration of *S. aureus* was prepared for use in the animal model.

### Animal Experimental

Thirty-six New Zealand white rabbits aged 6 months (with an average weight of 2.5–3.0 kg) were purchased from HFK Biosciences Experimental Animals Company (SCXK 2019-0008, China). Single cage feeding was employed under specific pathogen-free conditions. All experimental procedures were conducted according to the National Institutes of Health Guide for Care and Use of Laboratory Animals, and the protocol was approved by the Laboratory Animal Management Committee/Laboratory Animal Welfare Ethics Committee, Hebei University (Approval No. IACUC-2020002SR). Animals were housed with an ambient temperature of 22 ± 2°C, with relative air humidity of 40%–70%, and food and sterile water were supplied according to the experimental requirements.

### Determination of the Minimum Inhibitory Concentration and the Minimum Bactericidal Concentration

Experiments were performed in 96-well flat bottom plates. First, 20 μl of MRSA suspension and 180 μl of LD4 compound were added to each well. LD4 was prepared at concentrations of 2, 4, 8, 16, 32, 64, 128, 256, and 512 μM. The plate was then placed in the dark at 37°C for 30 min, then in the light for 30 min or in the dark again as a dark control sample. Then, the samples were further incubated in the dark at 37°C, and the number of colony-forming units (CFU) was evaluated after 18 h. Three groups of independent experiments were conducted (Rodrigues et al., 2013).

### Fractional Inhibitory Concentration Index

Gentamicin and LD4 were dissolved in ultrapure water to MIC concentrations of 4×, 2×, 1×, 0.5×, and 0.25×, which were then used to determine the fractional inhibitory concentration index (FICI). Using the chessboard design method, 50 μl of each drug at the different concentrations was added to the horizontal and vertical columns of a 96-well plate, respectively. Then, 100 CFU/ml of bacterial suspension (10^6^ CFU/ml) was added, with gentle mixing. The plates were stored in the dark at 37°C for 30 min, then placed in the light for 30 min. After illumination, the samples were incubated in the dark at 37°C for 16–20 h, and the MIC of each antibacterial agent in the combination was determined using the above method. For each combination of antibacterial drugs, we calculated the FICI by determining the ratio of the MIC of the combination divided by the MIC of each individual antibacterial drug, and then finding the sum of the two values (see Formula 2): FICI = [MIC_A(withB)_/MIC_A(alone)_] + [MIC_B(withA)_/MIC_B(alone)_]. The following criteria were used to interpret the FICI data: synergy, FICI ≤ 0.5; no interaction, FICI 0.5–4.0; antagonism, FICI > 4.0 (Barbee et al., 2014).

### Osteomyelitis Model

The rabbits were weighed and 3% pentobarbital solution (30 mg/kg) was injected into the ear margin vein. After anesthesia, the rabbits limbs were fixed in the supine position. The distal ends of the knee joints of the bilateral hind limbs were skinned and shaved to 5 cm from the distal ends of the knee joints, and sterile sheets were laid after iodophor disinfection. Then, 1 cm below the knee joint space in the anterior medial part of the upper tibia was taken as the incision starting point, and a longitudinal incision was made along the tibia. The skin was cut subcutaneously, and the fascia and muscle were separated layer by layer, the periosteum was peeled off, and the anterior medial part of the upper tibia was exposed. A 1 mm dental drill was used to drill into the medullary cavity. Then, approximately 0.2 ml of bone marrow was extracted with a 5 ml syringe, and 0.1 ml of sodium correlate solution was injected into the medullary cavity with a 1 ml needle. After 5 min, the solution was injected into the medullary cavity with a 1 ml syringe. In the same way, the contralateral hind limbs were modeled.

### Treatment of Osteomyelitis

Thirty-six New Zealand white rabbits were randomly divided into four groups: (a) control group without treatment, (b) an antibiotic treatment group, (c) PACT treatment group, and (d) PACT + antibiotic treatment group. For the PACT + antibiotic group, 0.2 ml of LD4 solution (40 μmoL) was injected locally into the wound, 0.4 ml of the antibiotic solution was injected intramuscularly, and laser irradiation was performed after 30 min in the dark. The irradiation duration was 15 min. The next day, the photosensitizer was injected, 0.4 ml of the antibiotic solution was injected intramuscularly and laser irradiation was performed for 15 min, all of which constituted one course of treatment. For the PACT group, the same steps were followed, except that no antibiotic solution was injected. For the antibiotic group, 0.2 ml of normal saline was injected into the injured area, and 0.4 ml of the antibiotic solution was injected intramuscularly every day. For the control group, 0.2 ml of saline was injected locally into the wound, and 0.4 ml saline was injected intramuscularly. The above treatments were repeated for seven courses, and the treatment lasted 2 weeks in total.

### Body Temperature and Weight

The weight of the white rabbits was recorded before, and every 5 days after, the operation, and the weight changes were calculated. The body temperature of the rabbits was measured with the electronic thermometer at 9:00 am before and after the procedure, the thermometer was inserted into the anus at a depth of 2.0–3.0 cm and the reading was recorded after 30 s.

### Gross Observation

After the operation, the rabbits were monitored in terms of their activity levels and eating habits. Exudation from the incision was also recorded, as well as the presence or absence of sinus formation and pus outflow. Mortality was recorded. The experimental animals were euthanized 2 or 5 weeks after treatment. The exposed tibia was entered along the original incision to observe the bone shape and surrounding soft tissue. Using Tang Hui’s method (Tang et al., 2009), a double-blind visual observation score was recorded: 0 points, no sign of infection; 1 point, a tiny amount of erythema on the skin surface; 2 points, erythema on the skin surface with thickening of the tibial shaft and sinus tract, or pus exudation; 3 points, severe bone resorption, abscess formation. A score of ≥2 was defined as an apparent infection.

### X-ray Detection

Before treatment, 2 weeks after treatment, and 5 weeks after treatment, X-ray images of the tibia were recorded to observe the condition of the tibia and for radiological analysis. The severity of osteomyelitis infection was graded by the improved Norden osteomyelitis scoring method (Norden et al., 1986), with 0–7 points representing the severity of osteomyelitis from no infection to the most severe infection. The improved Norden osteomyelitis score includes four indexes: dead bone formation, bone destruction, bone hyperplasia, and a soft tissue inflammatory mass shadow. Each index is graded according to three grades: yes, suspicious, and no, and the specific criteria are shown in Table 1.

**Table 1:** Norden osteomyelitis score.

	Have	Suspicious	No
Dead bone formation	3.0	1.5	0
Destruction of bone	2.0	1.0	0
Hyperosteogeny	1.0	0.5	0
Soft tissue shadow	1.0	0.5	0

### Bacterial Cultivation

Before treatment, 2 weeks after treatment, and 5 weeks after treatment, the experimental animals were euthanized, the tibia was separated and cut with the infected area as the center, the bone marrow was collected from the initial bone tunnel of the tibia, frozen, and stored in a sterile Eppendorf tube, and the bone marrow tissue was ground as soon as possible. The ground stock solution was diluted to 10 ml with sterile normal saline, and 1 ml of the diluted solution was evenly spread on a Petri dish and incubated at 37°C for 24 h before counting using the dilution counting method.

### Histological Observation

Before treatment, 2 weeks after treatment, and 5 weeks after treatment, the experimental animals were euthanized. The proximal tibia specimens were fixed in neutral formalin buffer solution, decalcified with 10% ethylenediamine tetraacetic acid, embedded in paraffin, and cut into 6 μm longitudinal sections. Sections were stained with hematoxylin and eosin (HE). Smeltzer’s scoring system contains four indexes that can be used for histopathological grading by evaluating acute bone inflammation, chronic inflammation, periosteal inflammation, and osteonecrosis. Each parameter is divided into five grades (0–4 points; Smeltzer et al., 1997). The sum of all of the histopathological parameters is the total histological score, with a total possible score of 16 points, and ≥8 points being defined as an apparent infection. Scoring was performed by an independent, double-blind researcher who scored all of the sections. Specific scoring criteria are shown in Table 2.

**Table 2:** Smeltzer’s histological score.

Score	Acute inflammation	Chronic inflammation	Periosteum inflammation	Osteonecrosis
0	No inflammation	No inflammation	No inflammation	No osteonecrosis
1	Mild inflammation, no intramedullary abscess	Mild inflammation, no obvious intramedullary fibrosis	Mild inflammation without formation of subperiosteal abscess	One necrotic focus, no dead bone formation
2	Moderate inflammation, no intramedullary abscess	Moderate to severe chronic inflammation, no obvious intramedullary fibrosis	Moderate to severe inflammation without formation of subperiosteal abscess	Multiple necrotic foci, no dead bone formation
3	Mild inflammation with intramedullary abscess	Mild inflammation with intramedullary fibrosis	Mild inflammation with subperiosteal abscess formation	Single dead bone formation
4	Severe inflammation with intramedullary abscess	Moderate to severe chronic inflammation with intramedullary fibrosis	Moderate to severe inflammation with subperiosteal abscess formation	Multiple dead bones formation

### Detection of Procalcitonin

Before modeling, before treatment and every week after treatment, blood samples were collected from the ear vein of the rabbits in each experimental group (2 ml). Serum samples were collected by centrifugation (3,000 r/min), and the extracted serum was stored in 1 ml sterile Eppendorf tubes at −20°C. The PCT content in the serum was determined using an ELISA detection kit.

### Detection of Toxicity and Side Effects

The organs (heart, liver, spleen, lung, and kidney) of the experimental animals were excised, weighed (accurate to 0.1 g) after removing the blood, and the organ index (organ mass/body weight) was calculated. Then, the corresponding tissues were excised, fixed in neutral formalin buffer solution, and embedded in paraffin, sliced and stained for 48 h. The sections were stained with HE.

### Statistical Analysis

SPSS19.0 (IBM, United States) statistical software was used for statistical analysis, and origin7.0 (Lab, United States) was used for charting the data. The data are expressed as the mean ± SD, and variance analysis of repeated data was used for comparisons among groups. Comparisons between different groups were also conducted using one-way ANOVA, and multiple comparisons were made using the least significant difference (LSD) *t*-test. *p* < 0.05 was considered statistically significant.

## Results

### Determination of the MIC and MBC

The MIC and MBC of LD4 for MRSA were studied. The bacterial suspension (10^6^ CFU/ml) was incubated with LD4 in the dark at 37°C for 30 min, then exposed to the light at 650 nm and 25 J/cm^2^. The concentration required to change the suspension from turbid to clear was the MIC. By contrast, a concentration of 5 or fewer colonies on the plate was the MBC. As shown in Table 3, LD4 was able to inactivate bacteria effectively in the light, with an MIC value of 4.0 μM and an MBC value of 8.0 μM for MRSA. Whereas the dark toxicity of LD4 was low, and the MIC and MBC values for MRSA were both more than 500 μM. The MBC value confirmed that LD4 has strong antibacterial activity against MRSA. Therefore, LD4 is an effective drug for treating osteomyelitis caused by drug-resistant *S. aureus*.

### Fractional Inhibitory Concentration Index

The measurement results of gentamicin combined with LD4-PACT are shown in Table 4. The average FICI of gentamicin combined with LD4-PACT in MRSA is 1.0, which indicates that this combination has additive effect.

**Table 3:** Minimum inhibitory concentration (MIC, μM) and minimum bactericidal concentration (MBC, μM) of methicillin-resistant *Staphylococcus aureus*.

LD4	Light toxicity		Dark toxicity	
	MIC	MBC	MIC	MBC
MRSA	4.0	8.0	500	>500

**Table 4:** FICI measurement results of gentamicin combined with LD4-PACT.

Bacteria	MIC (μg/ml)				FICI^▲^	Interpretation
	MIC_A_	MIC_A(with B)_	MIC_B_	MIC_B(with A)_		
MRSA	2.0 μg/ml	1.0 μg/ml	4.0 μM	2.0 μM	1.0	Additive effect

MIC_A_, gentamicin; MIC_B_, amino tetraphenyl porphyrin compound LD4, modified by a basic amino acid; MIC_A(withB)_, amino tetraphenyl porphyrin compound LD4, modified by gentamicin and basic amino acids; MIC_B(withA)_, amino tetraphenyl porphyrin compound LD4, modified by basic amino acids and gentamicin. The following criteria were used to interpret the fractional inhibitory concentration index (FICI^▲^) data: synergistic effect, FICI ≤ 0.5; additive effect, FICI 0.5–1.0; irrelevant effect, FICI 1.0–2.0; antagonistic effect, FICI ≥ 2.0.

### *In vivo* Experiment

For PACT experiments *in vivo*, a concentration equal to 5× or 10× MIC is usually selected as the therapeutic dose (Xu et al., 2016). On the basis of preliminary experiments, 40 μM LD4 was selected as the therapeutic dose in this study, which was 10× MIC. The antibacterial effect of LD4 *in vivo* was evaluated by analyzing various indexes.

### Body Temperature and Weight

**Figure 2:**
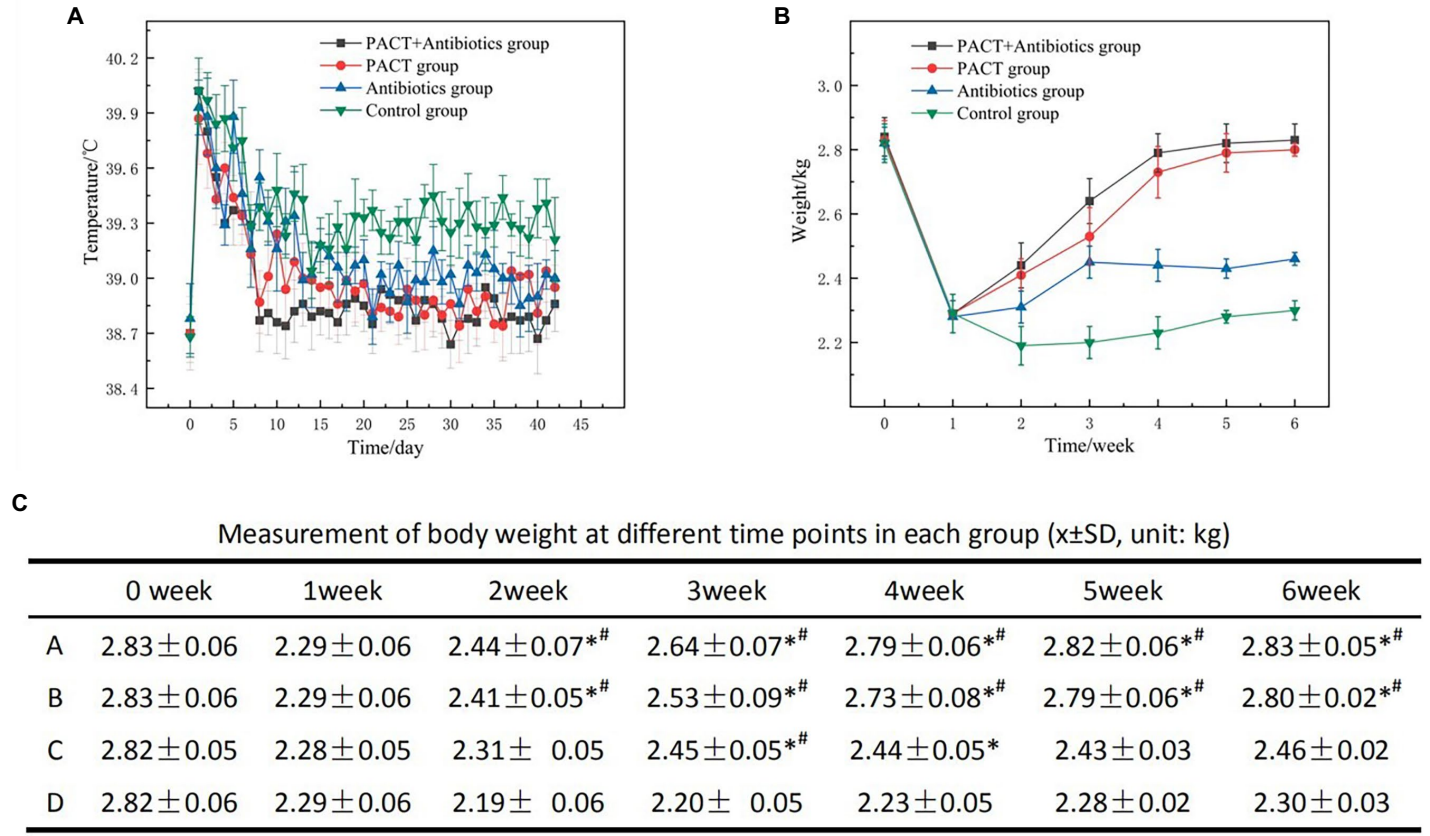
Data on body temperature and weight of the experimental animals. **(A)** Daily changes in body temperature in each group; **(B)** weekly changes in body weight in each group; and **(C)** the average and SD of body weight in each group, in which A, B, C, and D represent the PACT + GM group, PACT group, GM group, and control group, respectively; *p* < 0.05 indicates a statistically significant; *compared with the control group, # compared with the antibiotic (GM) group. GM, gentamicin.

The body temperature of each group of experimental animals was measured every day, starting from the day before modeling. Within 5 days after modeling, the body temperatures of animals in each group had increased obviously, but there was no significant difference between the groups (*p* > 0.05; Figure 2A). Compared with before treatment, after treatment, the body temperatures of the animals in each treatment group decreased obviously and gradually returned to normal. Compared with the control group, the body temperatures of animals in the PACT + antibiotic group, the PACT group, and the antibiotic group all decreased to different degrees, and this difference was statistically significant (*p* < 0.05), but there was no significant difference in body temperature changes among the treatment groups (*p* > 0.05).

The body weight of the experimental animals was measured before and after modeling, and the results were statistically analyzed (Figures 2B,C). After modeling, the weight of each group decreased significantly compared with that before modeling (*p* < 0.01). After the start of treatment, the weight of each group increased compared with that before treatment, especially in the PACT + GM group and the PACT group (*p* < 0.05). By contrast, the weight of the GM group increased slowly (*p* < 0.05). Compared with the control group, the weight changes in the PACT + GM group, PACT group and GM group were statistically significant (*p* < 0.05).

### Gross Observations

**Figure 3:**
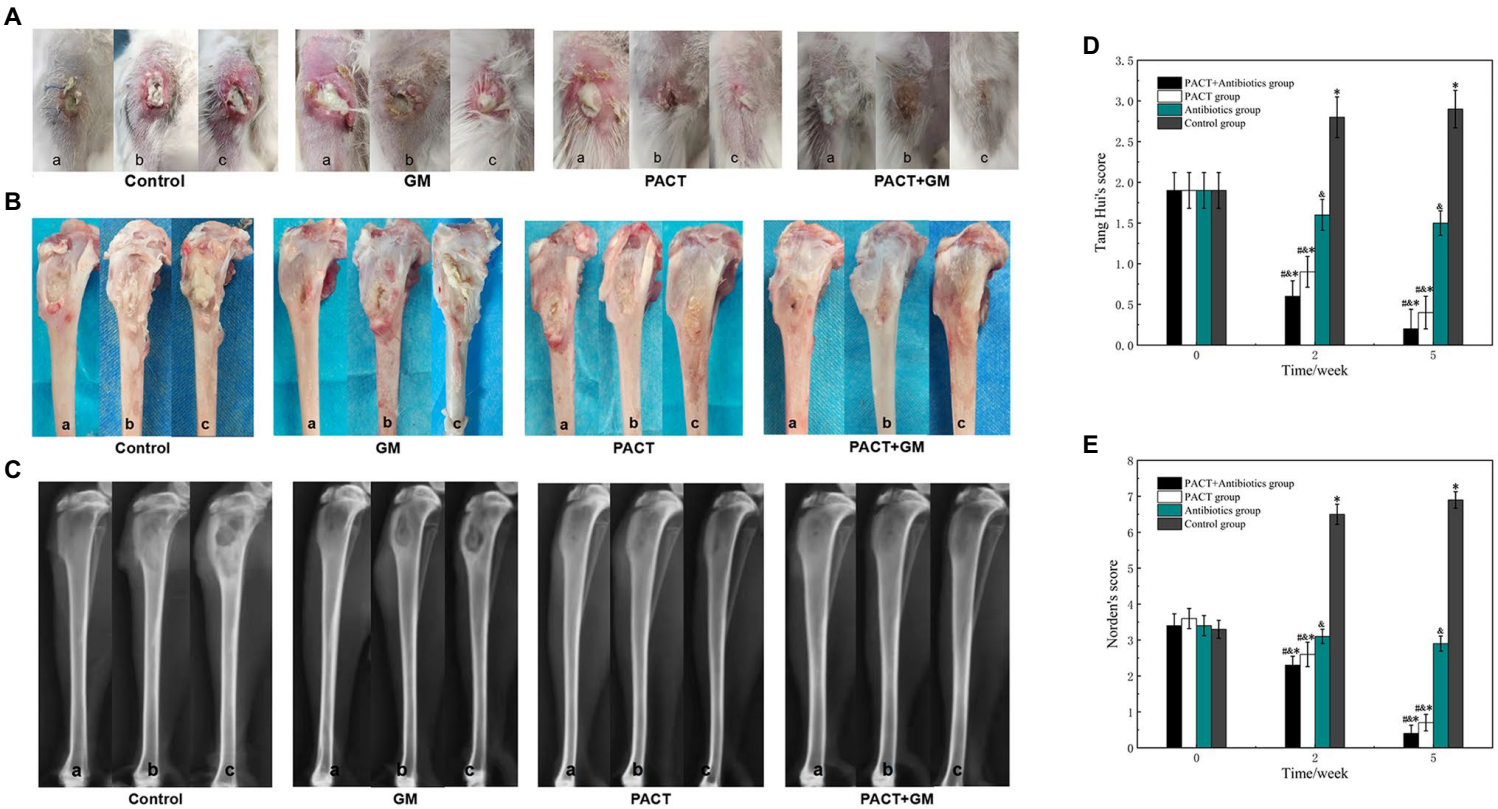
The results of visual observation and X-ray examination, A, B, and C samples represent before treatment, 2 weeks after treatment, and 3 weeks after treatment, respectively. **(A)** Macroscopic observation of the soft tissues in the four groups at different time periods; **(B)** macroscopic observation of the bone tissues in the four groups at different time periods; **(C)** X-ray images for the four groups at different time periods; **(D)** Scoring results from naked eye observations for each group; and **(E)** X-ray scores for each group. ^*^p < 0.05 indicates a statistically significant difference compared with before treatment, ^#^*p* < 0.05 indicates a statistically significant difference compared with the control group, and ^&^*p* < 0.05 indicates a statistically significant difference compared with the antibiotic group.

Visual observations can be used to directly evaluate the severity of osteomyelitis infection and the therapeutic effect. One week after modeling, all groups had noticeable swelling of the surrounding soft tissues and pus discharge from wounds, and some showed sinus formation (Figure 3A). After 2 weeks of treatment, swelling of the surrounding soft tissues, sinus formation, and bone destruction were observed in the control group. After 5 weeks of treatment, the degree of infection in the control group continued to worsen, with obvious bone destruction and dead bone formation. In the control group, the degree of soft tissue swelling, sinus formation, and bone destruction was more typical than before treatment, and the degree of infection increased with the increase in time. After 2 weeks of treatment, the degree of infection in the GM group showed no obvious change, but the degree of infection was significantly less than that of the control group (*p* < 0.05). After 5 weeks, the degree of infection in the GM group was significantly worse than that before treatment, but there was no significant difference between the GM group and the control group (*p* > 0.05). After 2 weeks, the wound infection and bone destruction in the PACT group and PACT + GM group were reduced compared with the control group, especially in the PACT + GM group (Figures 3A,B). Furthermore, in the PACT + GM group, there was no obvious bone destruction after 5 weeks of treatment, and osteomyelitis was generally resolving. Through quantitative analysis of soft tissue swelling, abscess formation, sinus formation, and bone destruction, statistical analysis of each group was carried out more systematically. There was no significant difference in the visual score of each group before treatment (*p* > 0.05). With increased time, the naked eye scores for the control group were significantly higher than those before treatment, and the naked eye scores for each treatment group were lower than those of the control group. Among the treatment groups, the reduction was most obvious in the PACT + GM group, with the groups ranking according to the naked eye score as follows: control group > GM group > PACT group > PACT + GM group (Figure 3D). Compared with before treatment, the visual score for the control group increased (*p* < 0.05), the score for the antibiotic group decreased slightly, and the visual scores for the PACT + GM group and the PACT group decreased significantly, especially for the PACT + GM group (*p* < 0.01).

### Imaging Observations

X-ray imaging is an effective method to evaluate the degree of infection in bone, and it has a certain value in evaluating bone destruction and bone hyperplasia caused by an inflammatory reaction. After 2 weeks of treatment, the destruction of bone, and swelling of the surrounding soft tissue could be seen in the control group. Compared with the control group, the degree of bone destruction and soft tissue swelling in the GM group was lower. Compared with the control group, there was no obvious destruction of bone in the PACT group or the PACT + GM group, and the degree of swelling of surrounding soft tissues in the PACT group was lower. After 5 weeks, bone destruction and absorption, thickening of the tibial shaft and swelling of the surrounding soft tissue were evident in the control group. In the GM group, bone destruction, hyperostosis, sclerosing agent, formation of dead bone, and obvious swelling of the surrounding soft tissue were evident. In the PACT group, hyperplasia and sclerosis of the bone was observed in some bones, but no obvious swelling of the surrounding soft tissues was detected. There was no obvious bone destruction, hyperplasia, or sclerosis in the PACT + GM group, and no obvious swelling of the surrounding soft tissues. The bone defects in the PACT + GM group tended to heal (Figure 3C). There was no significant difference in the Norden scores among the groups before treatment (*p* > 0.05). After 2 and 5 weeks, the Norden score for each treatment group was lower than that of the control group, with the reduction being the most obvious in the PACT + GM group, followed by the PACT group. Compared with before treatment, the Norden score in the control group was increased, and the score in the antibiotic group did not change significantly. The Norden scores for the PACT + GM group and the PACT group decreased significantly, and this reduction was most apparent in the PACT + GM group (Figure 3E).

### Bacterial Cultivation

**Figure 4:**
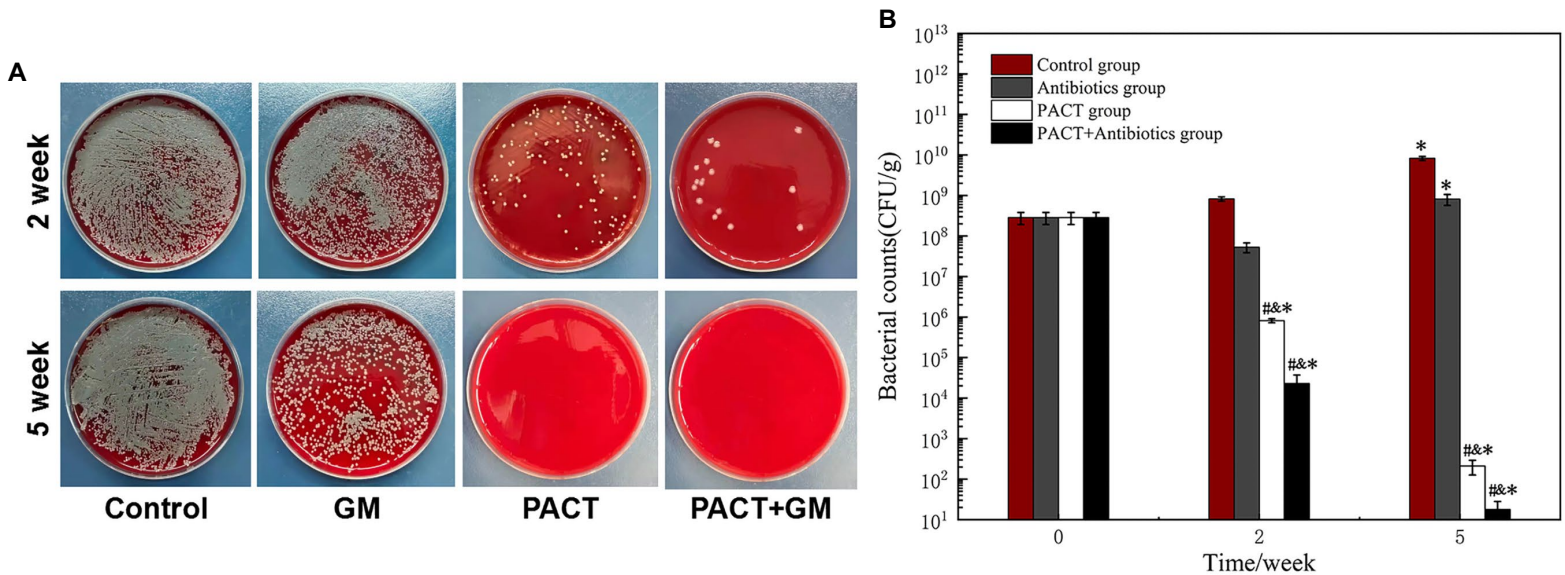
Results of bacterial culture of the bone marrow tissue from the bone marrow cavity. **(A)** Bacterial colonies from the bone marrow tissue of each group diluted to 1 × 10^−4^; **(B)** histogram comparing the numbers of bacterial colonies counted for each group. *p < 0.05 indicates a statistically significant difference compared with before treatment, ^#^*p* < 0.05 indicates a statistically significant difference compared with the control group, and ^&^*p* < 0.05 indicates a statistically significant difference compared with the antibiotic group.

A bacterial count is the gold standard method for the diagnosis of osteomyelitis, and it can also reflect the therapeutic effect. After 2 weeks of treatment, the number of colonies in the control group did not change significantly compared with before treatment, whereas the number of colonies in each treatment group was significantly decreased compared with before treatment. Among these groups, the number of colonies in the PACT + GM group decreased most obviously, followed by the PACT group (Figure 4A). After 5 weeks, the number of colonies in the control group increased slightly compared with that before treatment, and the number of colonies in the GM group increased slightly compared with that after 2 weeks of treatment. While in the PACT + GM group and the PACT group, the number of colonies decreased significantly compared with those before treatment (*p* < 0.01), with the numbers decreasing the most in the PACT + GM group, followed by the PACT group (Figure 4B).

### Histological Evaluation

**Figure 5:**
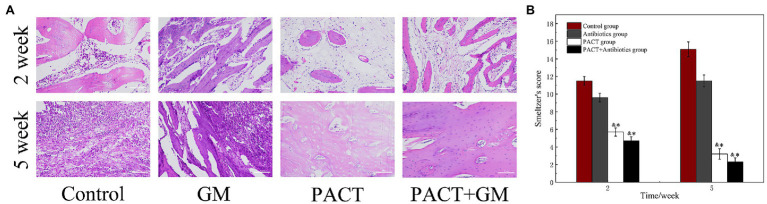
Results of histological evaluation. **(A)** HE staining of the bone tissues in different groups at different times and **(B)** Smeltzer scoring of the bone tissues in the different groups. *p < 0.05 indicates a statistically significant difference compared with the control group, and ^&^*p* < 0.05 indicates a statistically significant difference compared with the antibiotic group.

Histology can be used to evaluate the therapeutic effect of PACT on osteomyelitis at the cellular and molecular level. The experimental results showed obvious intraosseous inflammation, destruction of the tibial epiphysis, and severe abscess formation in the control group. The GM group showed moderate inflammation of the bone and periosteum and mild bone destruction after 2 weeks of treatment. Whereas, the PACT group and PACT + GM group showed only slight inflammation of the bone and periosteum after 2 weeks of treatment, and necrosis was only detected around the bone tunnel, with no bone or joint destruction. After 5 weeks, obvious inflammation and destruction of the bone were observed in the control group and the antibiotic (GM) group, with a large number of infiltrating inflammatory cells around the bone tissue, and irregularly arranged osteoblasts in some specimens (Figure 5A). However, the number of inflammatory cells in the PACT group and the PACT + GM group was significantly lower than before. Bone defects were filled with new bone, regular bone cells, and sometimes new osteoblasts. By scoring the bone specimens of each group, the control group showed the highest Smeltzer score, while the PACT + GM group showed the lowest score. Compared with the control group and the GM group, the PACT + GM group and the PACT group showed obvious differences (*p* < 0.01; Figure 5B).

### Procalcitonin

**Figure 6:**
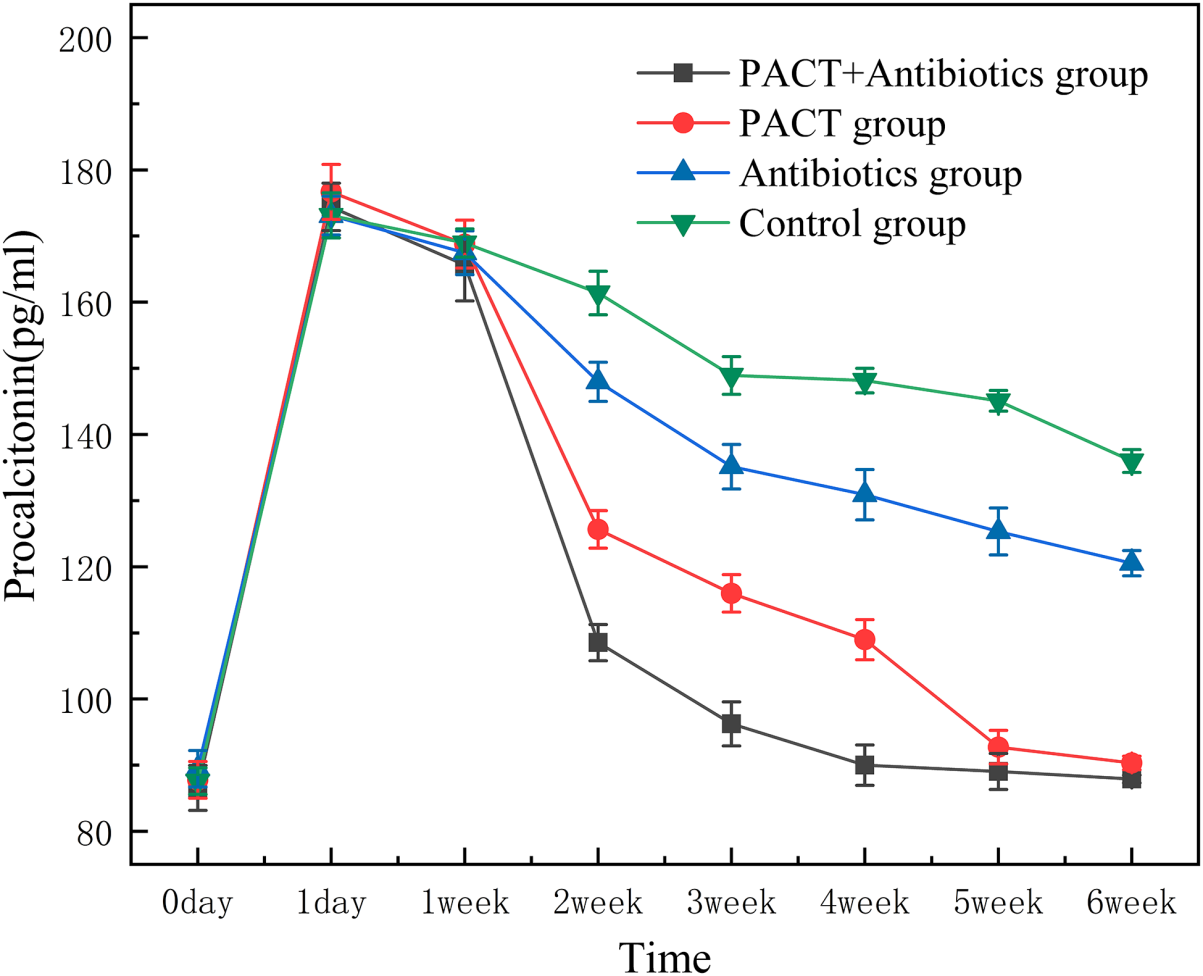
Detection results of serum procalcitonin.

PCT is an effective index to detect inflammatory reactions *in vivo*. The PCT content of each group before modeling was in the normal range and was significantly higher than before 1 day after modeling and 1 week after modeling, but there was no significant difference among the groups. With the prolongation of treatment time, the PCT content of each treatment group decreased significantly compared with the control group (*p* > 0.05), with the decrease being the most obvious in the PACT + GM group, followed by the PACT group (Figure 6). The contents of the PACT + GM group and the PACT group decreased with the increase in treatment time and remained normal before treatment. The PACT + GM group reached the normal level before therapy within 4 weeks, and the PACT group reached the normal level within 5 weeks. Although the content of PCT in the GM group also decreased with treatment time, it did not reach the normal level.

### Detection of Toxicity and Side Effects

**Figure 7:**
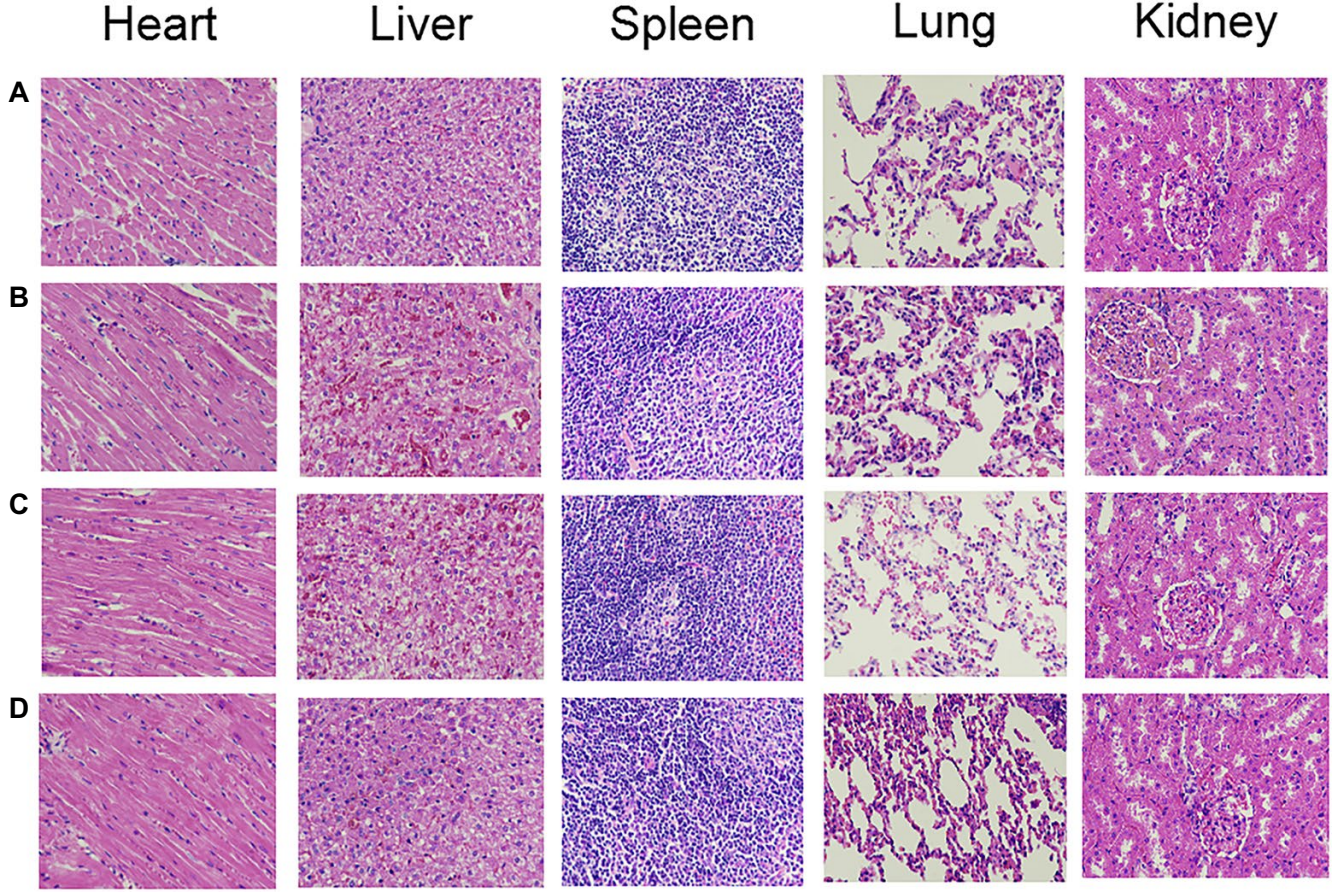
The HE staining results of organs in each group. **(A-D)** represent the control group, GM group, PACT group, and PACT + GM group, respectively.

Table 5 shows the mean and SD of the organ indexes for each group. Compared with the control group, there was no significant difference in the organ indexes among the PACT + GM group, PACT group and GM group (*p* > 0.05). No obvious tissue damage was found in the organs or tissues of the treatment groups or control group by HE staining (Figure 7), indicating that LD4, the new photosensitizer, has no obvious toxicity or side effects on animals.

**Table 5:** Organ index for each group (x ± SD, unit: ‰).

Group	Weight (kg)	Heart (mg/kg)	Liver (mg/kg)	Spleen (mg/kg)	Lung (mg/kg)	Kidney (mg/kg)
Control	2.8 ± 0.11	3.0 ± 0.10	41.8 ± 1.57	1.0 ± 0.15	6.1 ± 0.23	8.1 ± 0.17
GM	2.7 ± 0.18	3.1 ± 0.14	42.4 ± 2.05	1.1 ± 0.12	6.2 ± 0.32	8.2 ± 0.38
PACT	2.5 ± 0.09	3.0 ± 0.15	41.9 ± 2.03	1.0 ± 0.11	6.1 ± 0.36	8.2 ± 0.46
PACT + GM	2.4 ± 0.10	3.0 ± 0.10	41.2 ± 1.12	0.9 ± 0.10	6.0 ± 0.43	8.3 ± 0.33

*p* > 0.05, indicates that the difference was not statistically significant.

## Discussion

Although significant progress has been made in the treatment of osteomyelitis with advances in orthopedic surgery and antibiotic therapy, many challenges remain in the treatment of this type of serious infection. Radical surgical debridement is harmful to the body and rarely results in complete debridement. Antibiotic therapy includes long-term systemic antibiotic therapy and local antibiotic therapy with delivery devices made of biodegradable or non-biodegradable materials. Due to the increasing frequency of antibiotic use, the rapid adaptation of microorganisms to these drugs, and biofilm formation, bacteria gradually become resistant to these antibiotics (Hu et al., 2018). The Centers for Disease Control and Prevention (CDC) estimates that in the United States about 2 million people suffer from infectious diseases caused by multidrug-resistant bacteria every year, among which more than 23,000 people die (Solomon and Oliver, 2014). Bacterial biofilms readily form at bone infection sites. Because bacteria within a biofilm are shielded against attack from antibiotics, the host defense system, and the external environment, they are difficult to target even with a high concentration of antibacterial drugs, in some cases leading to persistent chronic infection of the bone in osteomyelitis patients.

In this study, we established an animal model of rabbit tibial osteomyelitis and used MRSA as the infecting bacterium. PACT alone or in combination with an antibiotic (PACT + GM) showed an excellent therapeutic effect in treating rabbit tibial osteomyelitis caused by MRSA. Observations 2 weeks after treatment revealed the presence of pus in the soft tissue around the tibia, sinus formation, and weight loss in the control group.

Compared with the control group, there was no evidence of pus and sinus formation in the wound of the PACT group or PACT + GM group, and weight gain was recorded. After 3 weeks of treatment, pus outflow and sinus formation were still evident in the soft tissues around the tibia of the control group, and body weight remained significantly lower than that before the experiment began (*p* < 0.01). Osteomyelitis in the PACT group and the PACT + GM group was essentially cured as wound healing was observed and body weight had returned to the pre-study level (*p* > 0.05).

X-ray imaging is a direct and effective method to evaluate osteomyelitis (Gariani et al., 2021). After 5 weeks, obvious bone destruction and absorption, thickening of the tibial shaft, and swelling of the surrounding soft tissue were evident in the control group; however, no evident bone destruction or dead bone tissue was found in the PACT group or the PACT + GM group. After 3 weeks of treatment, the amount of bone destruction increased, and large-scale dead bone tissue was evident in the control group, but there was no evident bone destruction or soft tissue swelling in the PACT group or the PACT + GM group, and this difference was statistically significant (*p* < 0.01). These results indicated that PACT and PACT combined with an antibiotic has a specific effect on osteomyelitis. Similarly, the results of bone marrow bacterial culture revealed that the numbers of bacteria in the PACT group and the PACT + GM group were significantly lower than in the control group (*p* < 0.01). This showed that PACT and PACT + GM can inactivate the pathogenic bacteria in the medullary cavity, lessening the likelihood of relapse after treatment. After the end of PACT, the number of bacteria in the medullary cavity continues to decrease, which may stimulate the body’s immunity and increase the body’s sensitivity to inflammatory factors (Xie et al., 2019). From the histopathological results, it can be seen that the numbers of osteoblasts in the PACT group and PACT + GM group are significantly higher than that in the control group (*p* < 0.05), which indicates that PACT can promote the differentiation of osteoblasts and is beneficial to the repair and healing of bone tissue (Ates et al., 2018). Taken together, the experimental results show that PACT combined with a low-dose antibiotic is a feasible treatment strategy for osteomyelitis, offering effective sterilization while promoting the healing of infected bone tissue.

PCT is an acute-phase protein with a kinetic speed that is faster than that of C-reactive protein and a high erythrocyte sedimentation rate (Velissaris et al., 2018). PCT is a sensitive and specific marker of many bacterial infections and is used to diagnose many illnesses, including osteomyelitis in diabetic patients (Schneider and Lam, 2007). It is also a potential tool to assess the severity of diseases (Delevaux et al., 2003). PCT and C-reactive protein are significantly correlated with the severity of bone infection, but only PCT can distinguish whether it is a complex infection (Vangaveti et al., 2021). A systematic review and analysis have shown that serum PCT is a sensitive detection method for diagnosing adult osteoarticular infection (Ertugrul et al., 2009; Ritchie et al., 2020). In the current study, PACT and PACT combined with an antibiotic obviously reduced the PCT content in serum and alleviated the inflammatory reaction caused by osteomyelitis.

The three essential elements of PACT are a photosensitizer, an excitation source, and oxygen. When working together, they produce obvious cytotoxicity, but none of these elements alone exerts an obvious therapeutic effect. Among them, the photosensitizer is the key factor affecting the efficacy of PACT. The new photosensitizer LD4 can selectively target bacterial pathogens, destroying bacterial cell walls and cell membranes and leading to genomic DNA damage (Xu et al., 2016). In *in vivo* experiments, photosensitizer LD4 showed a pronounced bactericidal effect on wounds infected with a mixture of gram-positive and gram-negative bacteria (Xu et al., 2016). However, to date, research on the treatment of osteomyelitis by LD4-PACT has not been reported. In this study, we found that LD4-PACT had a noticeable bactericidal effect on osteomyelitis. In addition, it can inhibit the secretion of inflammatory factors and promote the repair and healing of bone tissue, providing a new treatment method for osteomyelitis. We also evaluated the organ index and analyzed the organs of each group histologically. There was no significant statistical difference between the organ index and the histology of each group (*p* > 0.05), which indicated that the photosensitizer LD4 had no apparent toxic side effects on normal tissues and organs and therefore has potential for clinical use in the treatment of osteomyelitis patients.

In a previous study (Salehpour et al., 2019) researchers determined the penetration depth of visible light and near-infrared light into bone tissue and found that the while both penetrate bone tissue, the depth of penetration depends on the bone type and the region. Studies have also shown that porphyrin photosensitizer, excited by a laser at a wavelength of 635 nm, has good therapeutic effects against osteomyelitis (Bisland et al., 2006), The absorption spectrum of photosensitizer LD4 used in this study showed that lasers with wavelengths of 650 nm and 445 nm can effectively activate porphyrin derivatives (Meng et al., 2015). The penetration depth into most tissues is greater with the 650 nm red laser than the 445 nm blue laser. Therefore, a 650 nm laser-mediated photosensitizer LD4 was used in our rabbit osteomyelitis model, and the results were analyzed according to gross observations, X-ray imaging, histology, and PCT levels. The results showed that PACT could alleviate the inflammatory reaction of osteomyelitis to a certain extent and promote the healing of bone tissue (Ates et al., 2018). In addition, PACT combined with a low-dose antibiotic showed an improved therapeutic effect on osteomyelitis.

Osteomyelitis is difficult to cure completely, and it usually requires systemic use of large doses of antibiotics to control the infection; however, such treatment is accompanied by many side effects. In this study, a low-dose antibiotic was used, minimizing the systemic side effects. From the results of gross observations, X-ray scores, PCT levels and histological examination, it was clear that the low-dose antibiotic reduced the infection and controlled the inflammation. After treatment stopped, the animals treated antibiotic alone showed a resurgence of infection, which indicated that antibiotics alone could not completely treat osteomyelitis. However, in the animals treated with PACT, the infection was obviously reduced and there was no recurrence or aggravation of infection after the treatment was stopped. An advantage of PACT therefore appears to be that the likelihood of recurrence of infection is low. As shown in our study, the combination of low-dose antibiotic therapy for systemic treatment and PACT for local treatment appears to be effective for the treatment of osteomyelitis.

The following limitations of our study should be considered. (a) A *S. aureus* strain was used, which is the most common causative agent of osteomyelitis. However, as a result of the increase in open fractures and the overuse of antibiotics, the proportion of infections caused by *S. aureus* is decreasing annually, while the infection rate of *P. aeruginosa* is increasing annually (Zhang et al., 2019). Our findings showed that PACT combined with antibiotic therapy is effective against *S. aureus*-induced osteomyelitis, but its efficacy against osteomyelitis induced by *P. aeruginosa* and other bacteria remains unclear, and further research is needed to confirm this. (b) At present, the common types of osteomyelitis are post-traumatic osteomyelitis, osteomyelitis after prosthesis implantation, and diabetes-related osteomyelitis (Aicale et al., 2020). However, the preparation of the osteomyelitis model in this experiment was relatively simple and may not be fully representative of these different types of osteomyelitis. Therefore, in future studies, models that correspond to specific osteomyelitis types may be needed prior to clinical application.

## Conclusion

This study confirmed the feasibility and effectiveness of LD4-mediated PACT in treating osteomyelitis caused by drug-resistant bacteria. LD4-mediated PACT was effective at eliminating drug-resistant *S. aureus* and inhibiting the secretion of inflammatory factors, thereby promoting the healing of bone tissue. Furthermore, our findings showed that the combination of PACT with antibiotics had a specific synergistic effect in the treatment of osteomyelitis. This may allow for a reduction in the dosage of antibiotics and provides a promising new method for future clinical research into the treatment of osteomyelitis.

## Data Availability Statement

The original contributions presented in the study are included in the article/Supplementary Material; further inquiries can be directed to the corresponding authors.

## Ethics Statement

The animal study was reviewed and approved by the Experimental Animal Management Committee/Experimental Animal Welfare Ethics Committee of Hebei University.

## Author Contributions

XY, designing experimental scheme, leading animal experiments, writing articles, and analyzing statistical data, etc. ZF, histopathological analysis. YF, participate in animal experiments and draw flow charts. LZ and JP, involved in animal experiments. TL, provide photosensitizer for the experiments. ZZ, guiding animal experiments and writing articles. JZ, guiding animal experiments. The photosensitizer LD4 in photodynamic therapy was provided by Professor LT. All authors contributed to the article and approved the submitted version.

## Funding

The support comes from the China National College Students’ Innovation and Entrepreneurship Training Program (202110075032), the Medical Science Cultivation Program of Hebei University (2021A03), the Science and Technology Plan Program of Baoding (2172P002), and Research Fund Project of Hebei Provincial Health Committee (20220034).

## Conflict of Interest

The authors declare that the research was conducted in the absence of any commercial or financial relationships that could be construed as a potential conflict of interest.

## Publisher’s Note

All claims expressed in this article are solely those of the authors and do not necessarily represent those of their affiliated organizations, or those of the publisher, the editors and the reviewers. Any product that may be evaluated in this article, or claim that may be made by its manufacturer, is not guaranteed or endorsed by the publisher.

## Supplementary Material

The Supplementary Material for this article can be found online at: https://www.frontiersin.org/articles/10.3389/fmicb.2022.876166/full#supplementary-material

Supplementary Table S1 Results of drug sensitivity test of MRSA.

Supplementary Figure S1 Chemical structural formula of LD4.
