# Supplementary material for: Antimicrobial Photodynamic Therapy Involving a Novel Photosensitizer Combined With an Antibiotic in the Treatment of Rabbit Tibial Osteomyelitis Caused by Drug-Resistant Bacteria
Source: Front Microbiol. 2022 Apr 22;13:876166. doi: 10.3389/fmicb.2022.876166 (PMC9073078; doi:10.3389/fmicb.2022.876166)
Supplement: Supplementary Table S1 — Results of drug sensitivity test of MRSA. [file Table_1.DOC]

Antimicrobial spectrum of drug-resistant Staphylococcus aureus

| Antibiotic | MIC | Sensitivity |
| --- | --- | --- |
| Penicillin | >8 | R |
| Oxacillin | >4 | R |
| Cephalolin | <=0.5 | S |
| Gentamicin | <=2 | S |
| Daptomycin | <=1 | S |
| Clindamycin | <=0.25 | S |
| Erythromycin | <=0.25 | S |
| Ciprofloxacin | <=0.5 | S |
| Levofloxacin | <=1 | S |
| Moxifloxacin | <=0.25 | S |
| Vancomycin | =1 | S |
| Teicoplanin | <=1 | S |
| Tetracycline | <=0.5 | S |
| Minocycline | <=1 | S |
| Tigecycline | <=0.25 | S |
| Compound sulfanilamide | <=0.5/9.5 | S |
| Rifampin | <=0.5 | S |
| Linezolid | =2 | S |
| Chloromycetin | =8 | S |
