# Supplementary figures and images for: Antimicrobial Photodynamic Therapy Involving a Novel Photosensitizer Combined With an Antibiotic in the Treatment of Rabbit Tibial Osteomyelitis Caused by Drug-Resistant Bacteria
Source: Front Microbiol. 2022 Apr 22;13:876166. doi: 10.3389/fmicb.2022.876166 (PMC9073078; doi:10.3389/fmicb.2022.876166)

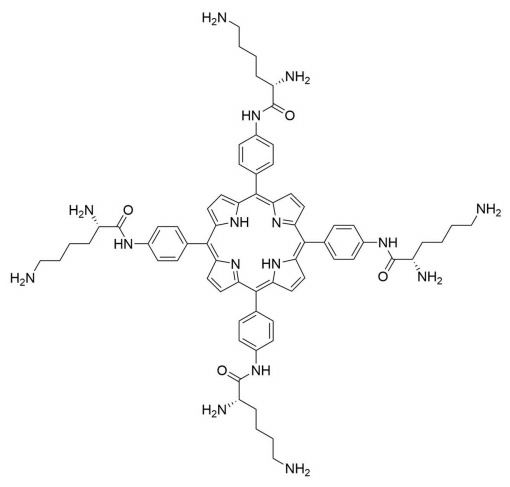

Supplement: Supplementary Figure S1 — Chemical structural formula of LD4. [file Image_1.TIF]
